# Performance of HR-pQCT, DXA, and FRAX in the discrimination of asymptomatic vertebral fracture in postmenopausal Chinese women

**DOI:** 10.1007/s11657-021-00939-0

**Published:** 2021-09-04

**Authors:** Meiling Huang, Vivian Wing-yin Hung, Tsz Kiu Li, Sheung Wai Law, Yulong Wang, Shangjie Chen, Ling Qin

**Affiliations:** 1 The Second School of Clinical Medicine, Southern Medical University, Guangzhou, China; 2 Bone Quality and Health Centre, Department of Orthopaedics and Traumatology, The Chinese University of Hong Kong, Hong Kong, China; 3 Department of Rehabilitation, Shenzhen Second People’s Hospital, The First Affiliated Hospital of Shenzhen University, Shenzhen, China; 4 Department of Rehabilitation, Shenzhen Baoan Hospital Affiliated to Southern Medical University, Shenzhen, China

**Keywords:** HR-pQCT, Asymptomatic vertebral fracture, Postmenopausal women, DXA, FRAX

## Abstract

***Summary*:**

Volumetric bone density (vBMD) and trabecular microarchitecture measured by high-resolution peripheral quantitative computed tomography (HR-pQCT) can discriminate the patients with high risk of asymptomatic vertebral fracture (VF) in postmenopausal Chinese women. These findings suggested that HR-pQCT could provide additional information on bone quality of the patients with asymptomatic VF.

**Introduction:**

Although there were several studies using HR-pQCT to investigate asymptomatic VF, it remains uncertain if HR-pQCT parameters can discriminate asymptomatic VF patients, especially in Chinese population. The purpose of this study was to investigate whether bone quality measured by HR-pQCT could discriminate asymptomatic VF independent of hip areal bone mineral density (aBMD) measured by dual-energy x-ray absorptiometry (DXA) and fracture risks evaluated using built-in Fracture Risk Assessment Tool (FRAX_BMD_).

**Methods:**

This is a nested case–control study. One hundred seventy-five ambulatory Chinese postmenopausal women aged 60–79 years were retrieved from Normative Reference Standards (NRS) cohort in Hong Kong. DXA was used to identify VF from lateral spine images (VFA) using Genant’s semi-quantitative method. Major osteoporotic fracture risk was calculated using FRAX tool. HR-pQCT was used to assess vBMD, microarchitecture, and estimated strength at both distal radius and tibia. Comparison of HR-pQCT parameters between asymptomatic VF and control was performed using covariance analysis. Logistic regression analysis was performed for calculating the adjusted odds ratio (OR) with 95% confidence intervals (CI) of fracture status as per SD decrease in HR-pQCT parameters.

**Results:**

Women with asymptomatic VF were older than those of the control in our NRS cohort. Nevertheless, after adjusted for covariance, asymptomatic VF showed significantly lower trabecular vBMD (Tb.vBMD) at radius but higher SMI at tibia as compared with those of the control. Tb.vBMD at radius yielded the highest value of area under the curve (AUC) as compared with total hip aBMD and FRAX_BMD_. However, no significant difference was found among each other.

**Conclusion:**

Tb.vBMD at the radius and SMI at the tibia provided by HR-pQCT can discriminate asymptomatic VF independent of hip aBMD and FRAX_BMD_ by DXA in postmenopausal women.

## Introduction

Osteoporotic fracture is a serious health problem affecting aging population worldwide. Its complications may lead to the risk of mobility reduction, increased cost of care, and even mortality [1]. Vertebral fracture (VF) is the most common type of osteoporotic fractures, especially in women with primary osteoporosis. Majority of patients with VF are asymptomatic, and therefore, early detection or diagnosis of VF is of great clinical significance. Dual-energy x-ray absorptiometry (DXA), conventional radiography, computed tomography (CT), and magnetic resonance imaging (MRI) can all be used to assess VF. CT and MRI techniques are still the major research tools for evaluating metabolic bone dysfunction, while the application of DXA can be extended beyond bone density measurement to assess VF and predict the 10-year fracture risk using FRAX [2]. Due to low costs and radiation dose, vertebral fracture assessment (VFA) using DXA becomes the most widely employed technique for diagnostic and serial monitoring of VF. VF can be identified by defining the morphology of vertebrae from thoracic to lumbar region of the spine (T4 to L4) semi-quantitatively in the VFA images [3].

Bone mineral density (BMD) measured by DXA can be used to predict VF. Previous studies showed that using areal BMD (aBMD) measured by DXA alone, only 41% of VF patient had T-score of BMD below −2.5 [4]. Recently, FRAX score, derived from femoral neck BMD (FRAX_BMD_) by DXA and clinical risk factors, has been used to predict 10-year risk of major osteoporotic and hip fractures [5]. However, it still underestimates the risk of osteoporotic fractures [6]. On the other hand, both the decrease of BMD and deterioration of bone microstructure attribute to the increase of bone fragility [7]; hence, bone quality such as cortical and trabecular architecture may also play an important role in fracture risk prediction [8, 9].

High-resolution peripheral quantitative computed tomography (HR-pQCT) is a non-invasive 3D imaging technology that enables quantitative measurement of volumetric BMD (vBMD) and trabecular microarchitecture, with high precision and relatively low-dose radiation. Furthermore, the HR-pQCT images can be used to estimate bone strength using finite element analysis (FEA) [10]. Bone microarchitecture has been shown to be an important parameter which reflects the mechanical properties of vertebral bodies [11]. In previous prospective studies, deterioration of trabecular and cortical bone and decrease in bone strength measured by HR-pQCT improve the prediction of low trauma or different kinds of fracture beyond aBMD or FRAX alone [12, 13], yet very few studies focus on asymptomatic VF. Asymptomatic VF is regarded the earliest osteoporotic fracture; early diagnosis and intervention of asymptomatic VF may prevent hip fractures that seriously affect the patients’ independence. Few studies report that there is a significant correlation between HR-pQCT parameters and the prevalence of asymptomatic VF in postmenopausal women [14]. Although it has been shown that bone microstructure parameters can predict the risk of osteoporosis and fracture independently of aBMD, it remains uncertain if HR-pQCT parameters can distinguish asymptomatic VF [15], particularly in Chinese population. In this nested case-control study, we aimed (1) to investigate the bone quality at distal radius and tibia in the patients with asymptomatic VF using HR-pQCT and (2) to examine whether HR-pQCT parameters could discriminate asymptomatic VF from the controls in Chinese postmenopausal women.

## Methods

### Participants and eligibility

This was a nested case–control study. All subjects in this study had participated in the Normative Reference Study (NRS), including 1072 ambulatory Chinese men (n = 544) and women (n = 528) aged 20 to 79 years in Hong Kong, a cohort study of the HR-pQCT normative reference dataset [16]. In the present study, all female participants aged over 60 were included (n = 175). This was a secondary analysis of NRS cohort data where all the assessments, including DXA and HR-pQCT measurements described below, have been conducted in the NRS cohort. All the study procedures and methods were approved by the Joint Chinese University of Hong Kong-New Territories East Cluster Clinical Research Ethics Committee (Ref: CRE.2014.310), and written consents were provided by all subjects. All subjects were able to walk independently. The same exclusion criteria were adopted as specified in the previous NRS cohort study [16].

### aBMD and vertebral fracture assessment

aBMD measurement at the proximal femur (i.e., femoral neck, trochanter, and total hip) and posterior-anterior lumbar spine (L1 to L4) was performed using DXA (Horizon; Hologic, Bedford, MA, USA). Standard operation procedure for scanning and analysis was performed by well-trained technicians to ensure the quality of the scans. Left hip was used for scanning unless there was a fracture, implant, or other conditions that could affect the accuracy of BMD evaluation. The coefficient of variation (CV) was used to express the short‐term precision error of the measurement of aBMD by DXA, which was 1.36% for the femoral neck, 1.19% for the total hip, and 1.01% for the lumbar spine in our center. According to the World Health Organization (WHO) diagnostic criteria, osteoporosis was defined as T-score ≤  − 2.5, osteopenia − 2.5 < T-score <  − 1, and normal T-score ≥  − 1. All subjects were classified according to the lowest T-score of the lumbar spine, femur neck, or total femur. FRAX_BMD_ score was also calculated using FRAX tool available in the DXA system using femoral neck aBMD for comparison.

VFA images were obtained by lateral spine scanning at T4-L4. Genant’s semi-quantitative (GSQ) assessment and morphologic analysis were used to classify VFA as follows: each VFA image was visually checked by a clinician (Zhu Y) to determine whether it contained any visualized vertebral fractures and was assigned a grade according to GSQ scale [17], where a reduction in vertebral height of 20–25% is classified as grade I (mild), a reduction of 25–40% as grade II (moderate), and a reduction of over 40% as grade III (severe). Identification of vertebrae was performed automatically by a built-in MXApro software. Only when the software could not correctly identify vertebral heights, an experienced radiologist was invited to adjust the positions of the six morphometry points manually. Participants who had grade I or above fractures, without symptoms such as back pain affecting the performance of daily activities or previous fragility fracture, were classified as the asymptomatic VF group, while those free of fractures were classified and assigned into the control group.

### Measurement of HR-pQCT

vBMD and microarchitecture of non-dominant distal radius and tibia were assessed by HR-pQCT (XtremeCT I, Scanco Medical AG, Switzerland). If the subject had a previous fracture at the scanning region, the non-fractured side was used for scanning and analysis. The standard scanning mode (60 kVp, 900 μA, 100 ms integration time, isotropic voxel size 82 μm) was used [18]. Subject’s forearm or leg was fastened in a carbon fiber casting in the scanner gantry. Reference line was set, and the scan was conducted 9.5 mm (distal radius) and 22.5 mm (distal tibia) proximal to the reference line. Motion artifact might occur during the image acquisition. In order to have good image quality for analysis, a visual grading system proposed by Pialat et al. was adopted. Image quality was divided into 5 levels: grade 1 (without motion artifact) to grade 5 (extreme motion artifacts) [19]. Images of grades 4 and 5 were excluded and not used for data analysis. All images were graded by a single operator after excluding motion artifact, where a total of 38 image datasets (26 radius and 12 tibia) were excluded from analysis in this study. Therefore, a total of 149 radius and 163 tibia images were finally used for statistical analysis.

The volume of interest (VOI) was automatically divided into cortical and trabecular components using a fully automated cortical compartment segmentation technique [20]. From the standard analysis, we obtained total and trabecular vBMD (Tot.vBMD and Tb.vBMD) in mg hydroxyapatite (HA/cm^3^). Trabecular number (Tb.N, mm^−1^) was determined using ridge-extraction method as the inverse mean spacing of the 3D ridges. Trabecular thickness (Tb.Th, mm) and trabecular separation (Tb.Sp, mm) were derived according to standard histomorphometrical methods. A fully automatic cortical compartment segmentation technique adapted from the method described by Burghardt et al. was used for the assessment of cortical compartment [20]. In this analysis, cortical vBMD (Ct.vBMD, mg HA/cm^3^) and cortical thickness (Ct.Th, mm) were obtained. Ct.Th was measured directly by removing the intracortical pores from the binary cortex image and using a distance transform. In addition, structure model index (SMI) and connectivity density (Conn.D, 1/mm^3^) were evaluated for each trabecular bone image [21]. SMI estimated the plate versus rod characteristics of trabecular bone [21], while Conn.D quantified trabecular connectivity by calculating the number of handles or closed loops in a trabecular network [22]. In our center, the short-term precision error (CV) of vBMD measurement (total, cortical, trabecular) was 0.38–1.03%, and that for all non-densitometric microstructural parameters was 0.80–3.73% [18].

### Estimated bone strength by micro-finite element analyses (μFEA)

μFEA analysis on the 3D images of the distal radius and tibia was performed using a built-in finite element solver software. A special stripping algorithm that specified a minimum Ct thickness of 6 voxels was used to recognize cortical and trabecular bone tissue. μFEA was performed by converting binary image data into isotropic brick element grid [23]. For all elements, a Poisson’s ratio of 0.3 and a Young’s modulus of 10 GPa were specified. The variables of FEA were stiffness (kN/mm) and the estimated failure load (Est.F.load, N). A uniaxial compression test with a 1000 N load was carried out. The apparent bone strength was estimated by stiffness (kN/mm). An estimate of failure load was calculated based on the assumption that bone failure would occur if more than 2% of the elements were strained beyond 0.7% strain [20].

### Patient demographics

Demographics including age, height, weight, a history of previous fracture and parent fracture hip, glucocorticoids, current smoking, rheumatoid arthritis, secondary osteoporosis, and alcohol consumption were recorded. Before DXA measurement, a wall-mounted stadiometer (SECA 240, Hamburg, Germany) was used to measure the standing height to closest 0.1 cm, and body weight was measured by a digital scale to nearest 0.1 kg. Body mass index (BMI) was calculated by dividing weight in kilograms by height in meters squared. Self-reported age at menopause was also recorded.

### Statistical analysis

SPSS (version 25.0, IBM, NY, USA) and MedCalc 19.1.3 (Ostend, Belgium) were used for statistical analysis. Mean ± SD was used to present all the continuous variables. Subjects were stratified into asymptomatic VF and control groups. According to the data type or the normal distribution test, Student’s t-test, Chi-square test, or non-parametric test was used to analyze differences between groups. HR-pQCT parameters and different severities of VF were compared by ANCOVA or Kruskal–Wallis test. Logistic regression models were performed to investigate which factors were independently associated with asymptomatic VF. Adjustment for age, BMI, total hip aBMD, or FRAX_BMD_ was performed in different models. These adjustments were used to examine whether the contribution of vBMD, microstructure, or strength parameters to fracture odds was independent of total hip aBMD and/or FRAX_BMD_. In the univariate analysis, the variables significantly (*p* < 0.05) associated with asymptomatic VF were included in the final logistic regression. The MedCalc 19.1.3 was employed for areas under the receiver-operating characteristic (ROC) curve analyses. The overall discriminative values of different risk scores were calculated. ROC curve analyses were performed to determine the optimal cutoff scores of HR-pQCT and DXA parameters for discriminating asymptomatic VF. The comparison of AUC between HR-pQCT and DXA parameters were performed with statistical significance set at *p*-value < 0.05.

## Results

### Characteristics of participants

In this nested case–control study, all 175 postmenopausal ambulatory Chinese women aged 60–79 years were included from NRS cohort. Table 1 represents the characteristics of study subjects. One hundred two subjects (58.3%) were defined as asymptomatic VF group using VFA. Among the asymptomatic VF patients, 61 (59.8%) had grade I fractures, and 41 (40.2%) were grade II or III fractures in at least one of the vertebrae. Seventy-three subjects (41.7%) without any vertebral fractures belonged to the control group. Both age and years since menopause in asymptomatic VF group were significantly higher than those of the control group (all *p* < 0.001). DXA results showed that in asymptomatic VF group, 19 (18.6%) women had normal BMD, 44 (43.1%) osteopenia, and 39 (38.2%) osteoporosis. Twenty-two (30.1%) women with normal BMD, 36 (49.3%) with osteopenia, and 15 (20.5%) with osteoporosis were in the control group. After adjustment for age, aBMD at femoral neck, total hip, and spine were generally lower in VF group, but only total hip and FRAX_BMD_ reached statistical significance as compared with the control group (*p* < 0.05). However, the other parameters, including body weight, body height, BMI, T-score at femoral neck, total hip, and lumbar spine, showed no significant difference between groups after adjustment for age (Table 1).

**Table 1:** Demographic characteristics of study subjects

Variables	Control (n = 73)	Asymptomatic VF (n = 102)	*p* value
Basic characteristics			
Age (years)^a^	66.66 ± 5.04	69.72 ± 5.09	**0.001**
Body weight (kg)^a^	56.24 ± 1.07	55.72 ± 0.89	0.713
Body height (m)^a^	1.53 ± 0.01	1.53 ± 0.01	0.977
Body mass index (kg/m^2^)^a^	23.95 ± 0.43	23.79 ± 0.36	0.786
Years since menopause^a^	16.04 ± 7.23	19.55 ± 7.09	0.001
DXA parameters			
T-score at femoral neck^a^	− 1.17 ± 0.13	− 1.46 ± 0.11	0.099
T-score at total hip^a^	− 0.07 ± 0.13	− 0.39 ± 0.11	0.055
T-score at lumbar spine^a^	− 1.00 ± 0.19	− 1.42 ± 0.16	0.110
Osteoporosis (%)^b^	15 (20.5%)	39 (38.2%)	
Osteopenia (%)^b^	36 (49.3%)	44 (43.1%)	
Normal BMD (%)^b^	22 (30.1%)	19 (18.6%)	
aBMD at femoral neck (g/cm^2^)^a^	0.66 ± 0.01	0.63 ± 0.11	0.098
aBMD at total hip (g/cm^2^)^a^	0.88 ± 0.01	0.84 ± 0.01	0.038*
aBMD at spine (g/cm^2^)^a^	0.88 ± 0.0.02	0.83 ± 0.01	0.074
FRAX_BMD_ (%)^a^	8.53 ± 0.67	10.50 ± 0.57	0.030*

Data as adjusted mean ± SD

^*^p < 0.05 after adjustment for age

^a^Independent t-test

^b^Chi-square test

### Lumbar spine T-score in patients with different severity of vertebral fracture

**Fig. 1:**
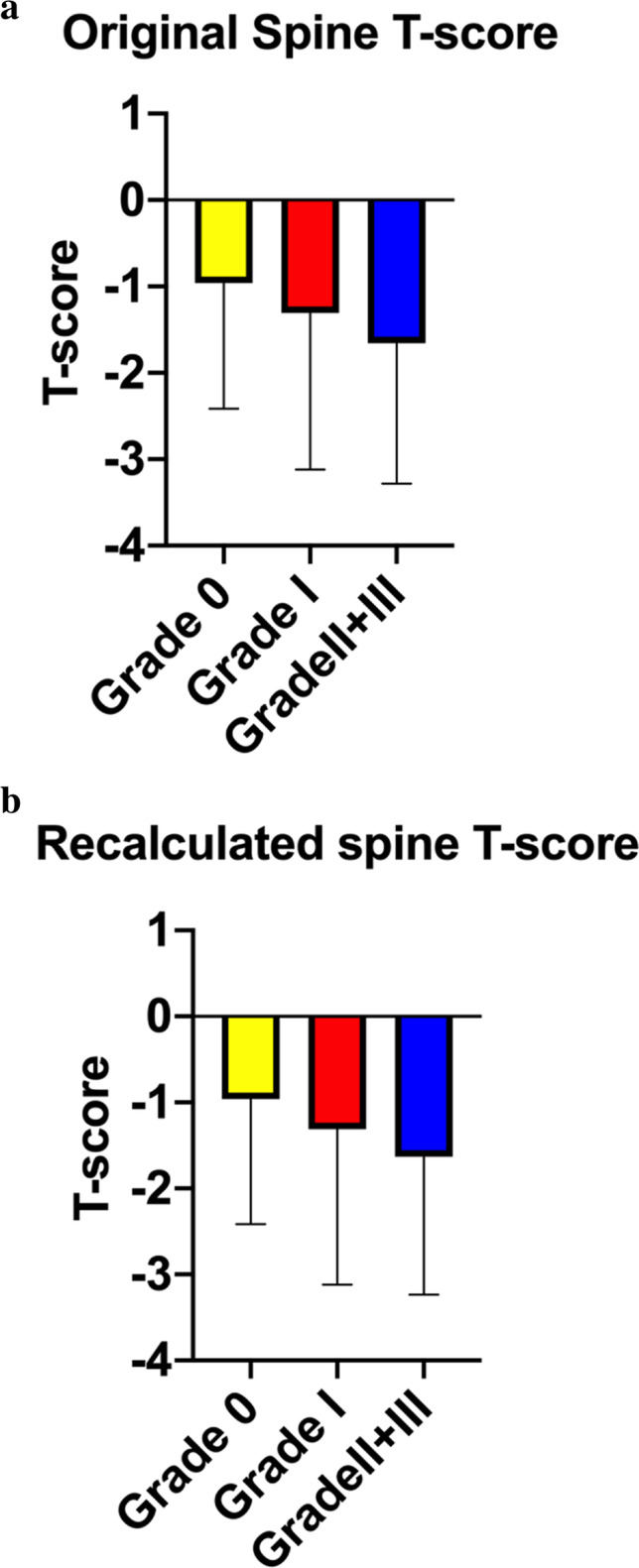
**a** Original BMD T-score trend in VFA vertebral grade. **b** Recalculated (excluding the lumbar spine fracture vertebrae) BMD T-score trend in VFA vertebral grade

The distribution of T-score in patients with the severity of vertebral fracture is shown in Fig. 1. The original spine T-score for grade 0, grade I, and grade II + III was − 0.959, − 1.308, and − 1.656, respectively (Fig. 1a). There was no statistical difference in T-score between groups (*p* = 0.086). This might be explained that aBMD falsely increased in the fractured vertebrae. In order to eliminate the influence of false-positive aBMD value in patients with vertebral fractures, the fractured vertebrae in L1-L4 were excluded, and the T-score was recalculated after the GSQ analysis (Fig. 1b). After excluding fractured vertebrae, however, the recalculated spine T-score remained similar to the original T-score in different VF gradings (Fig. 1b). Although the spine T-score declined with the vertebral grade deterioration, there was no significant difference found between two groups. Moreover, the recalculated spine T-score did not affect the number of patients with osteoporosis, osteopenia, or normal BMD.

**Fig. 2:**
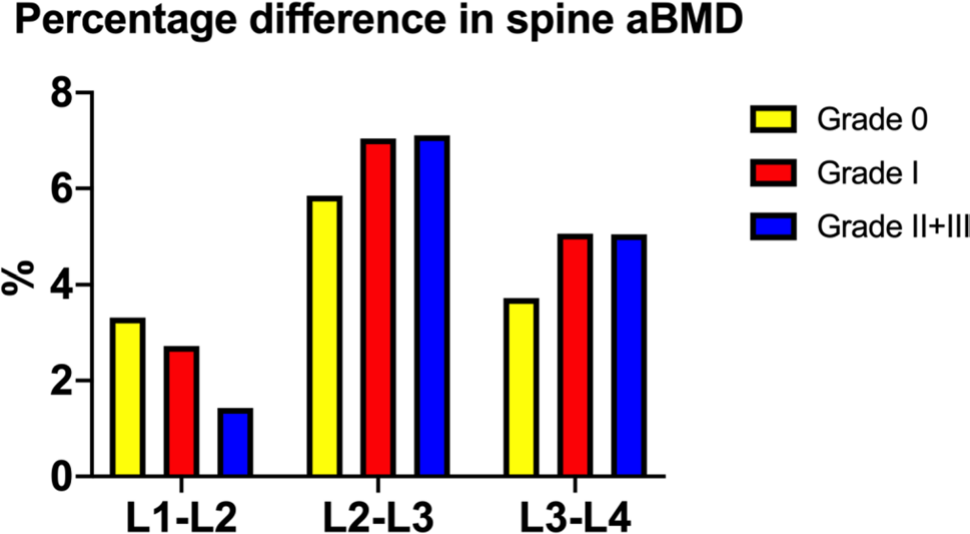
Percentage difference of the adjacent vertebrae in spine aBMD among asymptomatic VF grading

The percentage differences in spine aBMD at L1-L2 for grade 0 to grade II + III were 3.32%, 2.72% and 1.43%, respectively. The spine aBMD difference of L2-L3 and L3-L4 for grade I and grade II + III was higher than that of grade 0. In contrast, its difference was lower in L1-L2. The highest aBMD difference was found in L2-L3 in both groups (Fig. 2).

### Differences in vBMD, microarchitecture, and estimated bone strength

Considering the age difference between asymptomatic VF and the control group, adjustment of age was performed. The differences of vBMD, microarchitecture, and the estimated bone strength are presented in Table 2. Women with asymptomatic VF had significantly lower Tb.vBMD, Tb.N, Tb.Th, Conn.D, stiffness, and Est. F. load but higher Tb.Sp and SMI at distal radius (all *p* < 0.01). At distal tibia, women with asymptomatic VF had significantly lower Tot.vBMD, Tb.vBMD, Tb.N, Conn.D, stiffness, and Est.F. load but higher SMI, as compared with control group (all *p* < 0.05).

**Table 2:** Differences in volumetric bone mineral density (vBMD), bone microarchitecture, and estimated bone strength between women with asymptomatic vertebral fracture and the controls, with age as covariate

HR-pQCT variables	Distal radius			Distal tibia		
	Control (n = 65)	Asymptomatic VF (n = 84)	*p* value	Control (n = 68)	Asymptomatic VF (n = 95)	*p* value
Tot.vBMD, mgHA/cm^3^	296.07 ± 7.86*	227.45 ± 6.88*	0.082 ^a^	252.22 ± 5.67*	233.79 ± 4.63*	0.016 ^a^
Tb.vBMD, mgHA/cm^3^	123.48 ± 4.32*	104.77 ± 3.78*	0.002 ^a^	143.30 ± 3.86*	126.99 ± 3.24*	0.002 ^a^
Ct.vBMD, mgHA/cm^3^	841.10 (813.00, 898.25)	844.35 (812.78, 891.50)	0.595 ^b^	786.85 ± 6.32*	782.82 ± 5.31*	0.633 ^a^
Tb.N, 1/mm	1.43 (1.29, 1.69)	1.31 (1.11, 1.53)	0.004 ^b^	1.47 (1.34, 1.62)	1.34 (1.15, 1.53)	0.002 ^b^
Tb.Th, mm	0.07 (0.06, 0.08)	0.06 (0.05, 0.07)	0.006 ^b^	0.08 ± 0.01*	0.07 ± 0.01*	0.095 ^a^
Tb.Sp, mm	0.63 (0.52, 0.71)	0.69 (0.59, 0.84)	0.002 ^b^	0.59 (0.55, 0.67)	0.66 (0.58, 0.78)	0.085 ^b^
Ct.Th, mm	0.71 ± 0.02*	0.69 ± 0.02*	0.498 ^a^	0.93 ± 0.03*	0.89 ± 0.03*	0.295 ^a^
Conn.D, 1/mm^3^	2.59 ± 10.11*	2.15 ± 0.10*	0.005 ^a^	2.74 (2.26, 3.18)	2.38 (1.93, 2.88)	0.001 ^b^
SMI	2.09 ± 0.04*	2.24 ± 0.03*	0.004 ^a^	1.65 ± 0.04*	1.81 ± 0.03*	0.004 ^a^
Stiffness, kN/mm	57.59 (48.14, 63.22)	49.99 (45.02, 58.50)	0.007 ^b^	151.48 (138.51, 173.99)	142.61 (129.53, 158.45)	0.001 ^b^
Est. F. load, N	2866.01 (2455.32, 3139.50)	2529.53 (2283.28, 2951.44)	0.009 ^b^	7648.65 (7039.28, 8707.74)	7166.45 (6571.12, 7874.39)	0.001 ^b^

*Tot* total; *vBMD*, volumetric bone mineral density; *HA*, hydroxyapatite; *Tb*, trabecular; *Ct*, cortical; *Conn.D*, connectivity density; *SMI,* structure model index

^*^Data as adjusted mean ± SD

^a^Independent t-test

^b^Mann-Whitney U test. *p* < 0.05

The comparison of bone quality among different severities of vertebral fractures is summarized in Table 3. Significantly lower vBMD, deterioration of microarchitecture, and strength parameters were observed in the patients with asymptomatic VF at both radius and tibia. This was similar to the changes seen in the total cohort of both grade I and grade II + III as compared to grade 0 (Table 3), except Conn.D (*p* = 0.065), SMI (*p* = 0.096), stiffness (*p* = 0.077), and Est. F. load (*p* = 0.077) in the radius between grade II + III and grade 0, as well as Conn.D (*p* = 0.187) at the tibia between grade I and grade 0. However, there was significant differences found in Tb.N, Tb.Sp, and Conn.D in the tibia between grade II + III and grade I in the subgroup analysis.

**Table 3:** Differences in volumetric bone mineral density (vBMD), bone microarchitecture, and estimated bone strength between women in different Grade of VF

HR-pQCT variables	Grade 0	Grade I	Grade II + III	*p* value
Radius (n = 149)	N = 65	N = 51	N = 33	
Tot.vBMD,mgHA/cm^3 a^	295.82 ± 7.88	273.62 ± 8.69	283.85 ± 11.24	0.171
Tb.vBMD, mgHA/cm^3 b^	127.20 (99.45, 148.10)	106.00 (80.00, 129.60)*	87.00 (76.20, 128.80) *	0.001
Ct.vBMD, mgHA/cm^3 b^	841.10 (813.00, 898.25)	841.50 (809.60, 890.50)	853.30 (813.75, 892.90)	0.792
Tb.N, 1/mm^b^	1.43 (1.29, 1.69)	1.39 (1.15, 1.53) *	1.26 (1.09, 1.50) *	0.008
Tb.Th, mm ^a^	0.07 ± 0.01	0.06 ± 0.01	0.06 ± 0.01	0.100
Tb.Sp, mm^b^	0.63 (0.52, 0.71)	0.67 (0.57, 0.81) *	0.72 (0.59, 0.86) *	0.004
Ct.Th, mm^a^	0.71 ± 0.02	0.68 ± 0.02	0.73 ± 0.03	0.295
Conn.D, 1/mm^3 a^	2.59 ± 0.12	2.17 ± 0.13*	2.12 ± 0.17	0.019
SMI^a^	2.09 ± 0.04	2.24 ± 0.04*	2.24 ± 0.05	0.017
Stiffness, kN/mm^b^	57.59 (48.14, 63.22)	50.29 (45.17, 58.11) *	49.36 (44.80, 61.06)	0.024
Est. F. load, N	2866 (2445, 3139)	2546 (2293, 2546) *	2478 (2277, 3115)	0.034
Tibia (n = 163)	N = 68	N = 57	N = 38	
Tot.vBMD, mgHA/cm^3 a^	252.29 ± 5.69	234.67 ± 6.06	232.37 ± 7.75	0.053
Tb.vBMD, mgHA/cm^3 a^	143.55 ± 3.85	130.16 ± 4.10*	121.81 ± 5.23*	0.004
Ct.vBMD, mgHA/cm^3 a^	786.89 ± 6.35	783.39 ± 6.76	781.88 ± 8.64	0.884
Tb.N, 1/mm^b^	1.47 (1.34, 1.62)	1.38 (1.25, 1.64)	1.31 (1.14, 1.41) *^#^	0.001
Tb.Th, mm^a^	0.08 ± 0.01	0.07 ± 0.01	0.07 ± 0.02	0.249
Tb.Sp, mm^b^	0.59 (0.55, 0.67)	0.64 (0.54, 0.73)	0.69 (0.63, 0.82) *^#^	0.001
Ct.Th, mm^a^	0.93 ± 0.03	0.87 ± 0.03	0.90 ± 0.04	0.504
Conn.D, 1/mm^3b^	2.74 (2.26, 3.18)	2.46 (2.02, 3.14)	2.25 (1.73, 2.61) *^#^	0.003
SMI^b^	1.64 (1.45, 1.87)	1.89 (1.66, 2.04) *	1.78 (1.59, 2.08) *	0.005
Stiffness, kN/mm^b^	151.48 (138.51, 173.99)	144.54 (129.91, 159.61) *	140.52 (126.76, 152.09) *	0.003
Est. F. load, N^b^	7648 (7039, 8707)	7267 (6660, 8048) *	7143 (6481, 7715) *	0.003

*Tot*, total; *vBMD*, volumetric bone mineral density; *HA*, hydroxyapatite; *Tb*, trabecular; *Ct*, cortical; *Conn.D*, connectivity density; *SMI*, structure model index

^*^p < 0.05 compared with patients without VF (grade 0)

^#^*p* < 0.05 compared with patients with VF (grade I)

^a^ANCOVA (with Bonferroni correction for multiple testing)

^b^Kruskal-Wallis Test. *p* < 0.05

### Logistic regression and odds ratios (ORs)

Logistic regression results demonstrated that Tot.vBMD, Tb.vBMD, Tb.N, Tb.Sp, Conn.D, stiffness, Est. F.load, and SMI at both distal radius and tibia were associated with higher odds ratio of asymptomatic VF (Table 4).

**Table 4:** Crude and adjusted odds ratio (95% confidence interval) for per SD decrease of parameters of volumetric bone mineral density (vBMD), bone microarchitecture, and estimated bone strength (unless otherwise stipulated)

Variables	Crude		Model 1		Model 2		Model 3	
	OR (95%CI)	*p* value	OR (95%CI)	*p* value	OR (95%CI)	*p* value	OR (95%CI)	*p* value
Distal radius (n = 149)								
Tot.vBMD, mgHA/cm^3^	1.41 (1.02, 1.94)	0.033*	1.25 (0.89, 1.75)	0.203	1.10 (0.77, 1.57)	0.604	1.18 (0.83, 1.66)	0.359
Tb.vBMD, mgHA/cm^3^	1.95 (1.36, 2.79)	0.001**	1.82 (1.26, 2.63)	0.002**	1.64 (1.10, 2.45)	0.016*	1.68 (1.15, 2.45)	0.007**
Ct.vBMD, mgHA/cm^3^	1.21 (0.88, 1.67)	0.240	1.02 (0.71, 1.45)	0.935	0.92 (0.64, 1.33)	0.655	1.02 (0.72, 1.44)	1.02
Tb.N, 1/mm	1.54 (1.10, 2.17)	0.013*	1.40 (0.98, 2.00)	0.063	1.24 (0.85, 1.80)	0.258	1.28 (0.89, 1.85)	0.180
Tb.Th, µm	1.35 (1.04, 1.75)	0.024*	1.37 (1.05, 1.79)	0.022*	1.30 (0.98, 1.71)	0.066	1.35 (1.03, 1.77)	0.032*
Tb.Sp, mm	0.71 (0.49, 1.02)	0.065	0.78 (0.53, 1.14)	0.193	0.90 (0.61, 1.31)	0.569	0.86 (0.59, 1.26)	0.427
Ct.Th, µm	1.28 (0.94, 1.74)	0.118	1.12 (0.79, 1.56)	0.524	0.98 (0.69, 1.40)	0.928	1.08 (0.78, 1.50)	0.652
Conn.D, 1/mm^3^	1.55 (1.13, 2.13)	0.007**	1.43 (1.02, 1.99)	0.036*	1.29 (0.92, 1.82)	0.141	1.33 (0.95, 1.85)	0.095
SMI	0.64 (0.46, 0.89)	0.008**	0.66 (0.47, 0.93)	0.016*	0.71 (0.50, 1.01)	0.058	0.67 (0.48, 0.95)	0.026*
Stiffness, kN/mm	1.47 (1.07, 2.02)	0.018*	1.26 (0.88, 1.80)	0.205	1.05 (0.71, 1.57)	0.806	1.18 (0.83, 1.68)	0.360
F. load, N	1.39 (1.01, 1.92)	0.047*	1.18 (0.82, 1.70)	0.387	0.97 (0.64, 1.46)	0.877	1.10 (0.76, 1.57)	0.632
Distal tibia (n = 163)								
Tot.vBMD, mgHA/cm^3^	1.52 (1.11, 2.07)	0.008**	1.30 (0.92, 1.84)	0.134	1.12 (0.76, 1.66)	0.554	1.18 (0.82, 1.69)	0.378
Tb.vBMD, mgHA/cm^3^	2.18 (1.26, 3.78)	0.005**	2.05 (1.15, 3.66)	0.015*	1.73 (0.93, 3.21)	0.082	1.83 (1.01, 3.31)	0.045*
Ct.vBMD, mgHA/cm^3^	1.33 (0.98, 1.80)	0.070	1.14 (0.82, 1.58)	0.449	1.06 (0.75, 1.49)	0.761	1.12 (0.81, 1.55)	0.490
Tb.N, 1/mm	1.49 (1.07, 2.06)	0.018*	1.32 (0.93, 1.86)	0.121	1.14 (0.77, 1.68)	0.513	1.14 (0.79, 1.64)	0.480
Tb.Th, µm	1.17 (0.95, 1.45)	0.150	1.19 (0.94, 1.49)	0.144	1.16 (0.91, 1.46)	0.229	1.18 (0.93, 1.49)	0.179
Tb.Sp, mm	0.62 (0.39, 0.96)	0.034*	0.72 (0.46, 1.12)	0.140	0.84 (0.53, 1.32)	0.448	0.86 (0.55, 1.35)	0.509
Ct.Th, µm	1.35 (0.99, 1.83)	0.061	1.19 (0.85, 1.66)	0.320	1.09 (0.77, 1.54)	0.642	1.11 (0.79, 1.56)	0.551
Conn.D, 1/mm^3^	1.47 (1.08, 2.00)	0.016*	1.36 (0.98, 1.89)	0.066	1.22 (0.85, 1.74)	0.274	1.21 (0.87, 1.69)	0.247
SMI	0.56 (0.36, 0.88)	0.012*	0.53 (0.32, 0.86)	0.011*	0.56 (0.34, 0.93)	0.025*	0.57 (0.36, 0.92)	0.021*
Stiffness, kN/mm	1.63 (1.18, 2.25)	0.003**	1.43 (0.99, 2.05)	0.051	1.23 (0.79, 1.90)	0.363	1.24 (0.84, 1.81)	0.277
F. load, N	1.71 (1.23, 2.38)	0.001**	1.48 (1.02, 2.16)	0.041*	1.27 (0.80, 2.02)	0.320	1.30 (0.88, 1.93)	0.180

*Tot*, total; *vBMD*, volumetric bone mineral density; *OR*, odds ratio; *CI*, confidence interval; *HA*, hydroxyapatite; *Tb,* trabecular; *Ct*, cortical; *Conn.D*, connectivity density; *SMI*, structure model index

Model 1, adjusted for age + BMI; model 2, adjusted for age + BMI + total-hip aBMD; model 3, adjusted for FRAX_BMD_ alone

^*^p < 0.05, **p < 0.01

In model 1 (adjusted for age and BMI), Tb.vBMD, Tb.N, Tb.Th, Conn.D, and SMI at the distal radius and Tot. vBMD, Tb.vBMD, Tb.N, Conn.D, SMI, stiffness, and Est. F. load at the distal tibia remained significantly associated with higher odds ratio of VF (all *p* < 0.05). Further adjustment for age, BMI, and total hip aBMD (model 2), Tb.vBMD (OR 1.64, *p* = 0.016) at the distal radius and SMI (OR 0.56, *p* = 0.025) at the distal tibia remained significant in discriminating asymptomatic VF. Also, adjustment of FRAX_BMD_ alone in model 3, Tb.vBMD (OR 1.68, *p* = 0.007), Tb.Th (OR 1.35, *p* = 0.032), and SMI (OR 0.67, *p* = 0.026) at the distal radius and Tb.vBMD (OR 1.83, *p* = 0.045) and SMI (OR 0.57, *p* = 0.021) at the distal tibia were significantly associated with asymptomatic VF (Table 4). Furthermore, all these HR-pQCT variables at both radius and tibia after adjustment in model 3 were included in the final logistic regressions. Of all the significant variables, Tb.vBMD (OR 2.02, 95% CI 1.18 to 3.35, *p* = 0.010) at the distal radius showed the strongest association with asymptomatic VF.

### Area under ROC curve

Table 5 summarized the cutoff value and results of the ROC curve. Tb.vBMD at the radius showed the highest AUC (0.684) than total hip aBMD and FRAX_BMD_. The sensitivity of Tb.vBMD at radius, total hip aBMD, and FRAX_BMD_ were 86.2%, 69.9%, and 76.7% respectively, while the specificity of the three ROC curve was ranged from 42.65 to 60.8% (Table 5). However, there was no statistical significance in AUC of Tb.vBMD at radius, total hip aBMD, and FRAX_BMD_ (all *p* > 0.05).

**Table 5:** Results of the cutoff value and ROC curve

	Cutoffs	Sensitivity (%)	Specificity (%)	AUC	95% CI	AUC difference (*p* value)
Tb.vBMD_R (mgHA/cm^3^)	89.65	86.2	42.9	0.684	0.603–0.758	0.031 (0.526)^a^
total hip aBMD (g/cm^2^)	0.85	69.9	60.8	0.653	0.571–0.729	0.014 (0.733)^b^
FRAX_BMD_ (%)	9.25	76.7	52.0	0.667	0.585–0.742	0.017 (0.730)^c^

^a^Compared with total hip aBMD

^b^Compared with FRAX_BMD_

^c^Compared with Tb.vBMD_R (all *p* with Bonferroni correction)

Tb.vBMD_R: Tb.vBMD at radius, SMI_T: SMI at tibia

## Discussion

In this study, HR-pQCT parameters were shown to be able to discriminate asymptomatic VF independent of total hip aBMD and FRAX_BMD_ in Chinese postmenopausal women. Women with asymptomatic VF had significantly lower Tb.vBMD, Tb.N, Tb.Th, and Conn.D but higher SMI at distal radius than those of the controls. Lower Tot.vBMD, Tb.vBMD, Tb.N, Conn.D, stiffness, and Est.F.load but higher SMI at distal tibia were also found in asymptomatic VF group. Lower in Tb.vBMD at the radius and higher SMI at the tibia remained significant after adjustment for age, BMI, and total-hip aBMD. Tb.vBMD at radius yielded the highest value of AUC as compared with total hip aBMD and FRAX_BMD_. Our results illustrated that bone microarchitecture parameters measured by HR-pQCT were useful in discriminating the patients with asymptomatic VF, independent of DXA-derived total hip aBMD and FRAX_BMD._

vBMD, microarchitecture, and mechanical properties of bone measured by HR-pQCT play important roles in discriminating the patients with asymptomatic VF. DXA assesses aBMD on the lumbar vertebra, yet its 2D nature is not able to measure cortical and trabecular bone separately. The resolution of DXA scan only allows the measurement of the integral aBMD, but does not offer sufficient resolution for delineate microarchitecture of trabecular bone [8]. Central QCT provides quantitative volumetric measurement at spine. However, its cost and radiation dose are relatively high [24]. Hence, using central QCT to identify the patients with asymptomatic VF is not common in clinical practice. HR-pQCT is a peripheral device which provides high resolution images up to 61–82 µm with low radiation dose. HR-pQCT can also assess microarchitecture of bone and its estimated bone strength in addition to vBMD [25]. In previous prospective studies, deterioration of trabecular and cortical bone, and decrease of bone strength quantified by HR-pQCT improve the prediction power of incident fragility fractures as compared with aBMD alone by DXA [12, 13]. Our results showed that postmenopausal women with asymptomatic VF had lower Tb.vBMD at the radius but higher SMI at the tibia, as compared with the controls, after adjustment of age, BMI, and total-hip aBMD. Meanwhile, lower Tb.vBMD but higher SMI at both the radius and the tibia, and lower Tb.Th at the radius were found to be independent variables in discriminating asymptomatic VF as compared with the controls, after adjustment of FRAX_BMD_. Interestingly, trabecular density parameters showed strong association with asymptomatic VF, but not cortical parameters. It could be postulated that trabecular bone deterioration might contribute largely to the asymptomatic VF [26]. However, our results showed there were no significant differences in spine or femoral neck aBMD between asymptomatic VF patients and the controls.

To the best of our knowledge, there were few previous studies focused on using HR-pQCT for discriminating asymptomatic VF in Chinese women, although reports on using DXA to measure aBMD for predicting fracture risk were available [27]. Consistent with an early study [28], our findings showed that the subjects with VF had lower vBMD of radius and tibia. However, most of the previous studies focused on different types of fragility fracture in Caucasian women [12, 29, 30], including wrist [31], hip [32], and symptomatic vertebral fractures, but not asymptomatic ones in Chinese population [28, 30, 33]. Deterioration of trabecular and cortical bone microarchitecture at the distal radius and tibia was found in fractured patients when compared with non-fractures controls, after adjustment of aBMD in the hip or spine [33, 34], where for example, Vranken and co-workers [33] studied on Caucasian women aged 50–90 years old with a recent non-vertebral fracture and revealed the association of bone microarchitecture and bone strength with prevalent VF. This indicated that women with at least one prevalent vertebral fracture had significantly deteriorated total and trabecular vBMD at both tibia and radius.

In our study, Tb.vBMD at the distal radius and SMI at the distal tibia were found to be associated with higher odds ratio in asymptomatic VF, but not for Tot.vBMD and Ct.vBMD. The discrepancy of performance between trabeculae and cortical parameters might be explained by the fact that all of our subjects had asymptomatic VF, which was different from the previous studies on patients with symptomatic or prevalent VF. Pathologically, asymptomatic VF occurs in the early stages of osteoporosis. This period is mainly attributed to the degradation of trabeculae instead of cortical bone [35]. Furthermore, the previous studies with VF did not adjust for FRAX_BMD_ [28, 33]. In our current cohort, lower Tb.vBMD and higher SMI at both the radius and tibia, and lower Tb.Th at the radius were associated with increased VF odds ratio after adjusted for FRAX_BMD_. These findings were different from our previous study which studied on a group of older patients with hip fracture, where a significant loss of vBMD in trabecular bone became insignificant after adjustment of T-score at the femoral neck [32]. Zhu et al. reported that the peak incidence of hip fracture occurred at old age (75–80 years), at which there was already a remarkable bone loss. The measurement of decreased bone mass was well reflected by significant deterioration of bone microarchitecture, indicating bone fragility to a great extent. Hence, the added value of measuring the deterioration of bone microarchitecture would become very limited in old population, leading to inability to improve the prediction of fragility fractures [36]. In contrast, VF has two peaks of incidence: 55–60 and 70–75 years old [16]. In our current cohort, the mean age of asymptomatic VF was 69.7 ± 5.1 years; therefore, the changes in Tb.vBMD could be prominent after adjustment of hip aBMD and FRAX_BMD_.

Moreover, Tb.vBMD in radius was found to be a unique HR-pQCT parameter with the highest AUC of 0.684, which had a high discriminative power in identifying asymptomatic VF patients from those without fractures. Furthermore, lower Tb.vBMD at the radius in asymptomatic VF remained significant after adjustment of age, BMI, and total hip aBMD. Tb.vBMD at radius yielded the highest value of AUC as compared with total hip aBMD and FRAX_BMD_. However, the comparison of AUC among these variables showed no significant differences. Recent studies indicated that bone microstructure and the estimated bone strength were valuable in predicting the risk of fragility fractures in postmenopausal women, independent of aBMD [12, 30], which was superior to DXA and FRAX in fracture prediction [12]. However, longitudinal studies should be planned in the future to determine whether these parameters are of great value for VF prediction in asymptomatic patients.

Cortical porosity is associated with bone strength [37], and previous studies have shown an association between cortical porosity and prevalent fracture [38]. However, this study did not include cortical porosity in the analysis because of a 20% precision error in measuring cortical porosity [39], where at least a 40% difference was required to achieve a significant between-group difference. With advancing age, endocortical bones may gradually undergo trabeculation. Previous studies demonstrated that the active area of bone remodeling existed in the transitional zone between the cortical and trabecular bone [40, 41]. In our study, HR-pQCT (XtremeCT I) with a spatial resolution of 82 μm was used, where the spatial resolution was not high enough and the segmentation algorithm of cortical bone might lead to the inaccuracy in the calculation of cortical porosity. Hence, it might underestimate the cortical porosity but overestimating the trabecular thickness [41].

In our study, DXA T-score and aBMD were similar to a previous study of Torres et al. who reported that the majority of fragility fractures occurred in the subjects with osteopenia rather than osteoporosis as determined by aBMD using DXA [34]. Our study showed that approximately 38.2% of women with osteoporosis and 43.1% of women with osteopenia were identified as asymptomatic VF. Asymptomatic VF group had significantly lower aBMD at total hip but no significance at lumbar spine. Spine aBMD measured by DXA could be false positive as confounded by degeneration of vertebrae or aortic calcification in the older population. Therefore, the influence of vertebral fractures on aBMD should also be considered. In our study, after excluding the fractured vertebrae, the recalculated spine T-score declined when the vertebral grade deteriorated, yet such differences remained not significant between groups. Moreover, there was no difference in spine and femoral neck aBMD between VF and the controls after adjustment of age.

Tb.vBMD was associated with the severity of asymptomatic VF. There was a significant difference in Tb.vBMD at both radius and tibia measured by HR-pQCT and also in the subgroup analysis for severity of prevalent VF. Significantly lower Tb.vBMD was observed in grade I and grade II + III VF at both radius and the tibia than that of grade 0 VF, which confirmed the different sensitivity of HR-pQCT for different grades. This could be due to the different principles of the 2D and 3D techniques. DXA is a 2D scanning technique, which is used for obtaining aBMD during posteroanterior imaging of the lumbar spine, including the vertebral posterior elements (i.e., the spinous processes and pedicles). Lee et al. reported that the posterior elements accounted for 51.4 ± 4.2% of the total bone mineral content of the lumbar spine in DXA scanning [42]. On the other side, HR-pQCT is a 3D technique that enables the assessment of trabecular microarchitecture and estimates the bone strength of cortical bone and trabecular bone individually. DXA results showed that only 38.2% subjects had osteoporosis, but 43.1% of them had osteopenia in asymptomatic VF group. By WHO osteoporosis classification, DXA cannot identify all asymptomatic VF cases because 40–50% of them are osteopenia only. Therefore, HR-pQCT becomes an alternative yet advanced modality in discriminating asymptomatic VF patients. Interestingly, percentage difference in grades 0, I, and II + III of spine aBMD of the adjacent vertebrae was 3.32%, 2.72%, and 1.43% in L1-2; 5.85%, 7.04%, and 7.11% in L2-3; and 3.72%, 5.06%, and 5.04% in L3-4, respectively. This alerted us to pay attention to the patients when the percentage difference of spine aBMD was higher than 7% in L2-L3 and 5% in L3-L4.

There were a few limitations in this study. Firstly, our sample size might not be large enough in the current cross-sectional and nested study; large and follow-up study is desirable to confirm such predictive value in asymptomatic VF using HR-pQCT. We have not yet validated the model described in the current study; hence, future cohort is needed for model validation as this retrospective study had a time lag between the occurrence of fracture and bone measurement. Secondly, the diagnosis of VFs by VFA might be affected by some subjective factors, especially in grade I patients, by using Genant’s semi-quantitative (GSQ) approach and morphologic method for VFA classification [43]. Thirdly, volunteer nature might be a potential sampling bias that was controlled by stringent selection criteria and screening. Fourthly, HR-pQCT was used to evaluate the microstructure and vBMD of the peripheral bone rather than the spine. Currently, there is no imaging technology with high resolution and relatively low radiation available for central bone scanning, while HR-pQCT is the best imaging peripheral 3D technology to correlate with aBMD of the spine and hip [44]. Also, the single racial cohort limits the generalizability of our research, and caution should be paid to translate our results for other ethnicities.

In conclusion, the current study demonstrated that Tb.vBMD at the radius and SMI at the tibia were the most significant HR-pQCT parameters in discriminating patients with asymptomatic VF in postmenopausal Chinese women. Tb.vBMD at the radius was the most significant parameter associated with asymptomatic VF. HR-pQCT parameters help to identify asymptomatic VF from the patients who are at risk of fragility fractures. Future longitudinal study is warranted to evaluate whether HR-pQCT parameters could effectively predict osteoporotic fractures in Chinese population.

## References

[1] Kanis JA, Cooper C, Rizzoli R, Abrahamsen B, Al-Daghri NM, Brandi ML, Cannata-Andia J, Cortet B, Dimai HP, Ferrari S (2017). Identification and management of patients at increased risk of osteoporotic fracture: outcomes of an ESCEO expert consensus meeting. Osteoporos Int.

[2] Chou SH, LeBoff MS (2017). Vertebral imaging in the diagnosis of osteoporosis: a clinician’s perspective. Curr Osteoporos Rep.

[3] Genant HK, Li J, Wu CY, Shepherd JA (2000). Vertebral fractures in osteoporosis: a new method for clinical assessment. J Clin Densitom.

[4] Alacreu E, Moratal D, Arana E (2017). Opportunistic screening for osteoporosis by routine CT in Southern Europe. Osteoporos Int.

[5] El Maghraoui A, Sadni S, Jbili N, Rezqi A, Mounach A, Ghozlani I (2014). The discriminative ability of FRAX, the WHO algorithm, to identify women with prevalent asymptomatic vertebral fractures: a cross-sectional study. BMC Musculoskelet Disord.

[6] Holloway-Kew KL, Zhang Y, Betson AG, Anderson KB, Hans D, Hyde NK, Nicholson GC, Pocock NA, Kotowicz MA, Pasco JA (2019). How well do the FRAX (Australia) and Garvan calculators predict incident fractures? Data from the Geelong Osteoporosis Study. Osteoporos Int.

[7] Mao Y-F, Zhang Y, Li K, Wang L, Ma Y-M, Xiao W-L, Chen W-L, Zhang J-F, Yuan Q, Le N (2019). Discrimination of vertebral fragility fracture with lumbar spine bone mineral density measured by quantitative computed tomography. J Orthop Transl.

[8] Gallacher SJ, Gallagher AP, McQuillian C, Mitchell PJ, Dixon T (2007). The prevalence of vertebral fracture amongst patients presenting with non-vertebral fractures. Osteoporos Int.

[9] Du MM, Che-Nordin N, Ye PP, Qiu SW, Yan ZH, Wang YXJ (2020). Underreporting characteristics of osteoporotic vertebral fracture in back pain clinic patients of a tertiary hospital in China. J Orthop Translat.

[10] Vilayphiou N, Boutroy S, Sornay-Rendu E, Van Rietbergen B, Munoz F, Delmas PD, Chapurlat R (2010). Finite element analysis performed on radius and tibia HR-pQCT images and fragility fractures at all sites in postmenopausal women. Bone.

[11] Qiu S, Rao DS, Palnitkar S, Parfitt AM (2006). Independent and combined contributions of cancellous and cortical bone deficits to vertebral fracture risk in postmenopausal women. J Bone Miner Res.

[12] Biver E, Durosier-Izart C, Chevalley T, van Rietbergen B, Rizzoli R, Ferrari S (2018). Evaluation of radius microstructure and areal bone mineral density improves fracture prediction in postmenopausal women. J Bone Miner Res.

[13] Samelson EJ, Broe KE, Xu H, Yang L, Boyd S, Biver E, Szulc P, Adachi J, Amin S, Atkinson E (2019). Cortical and trabecular bone microarchitecture as an independent predictor of incident fracture risk in older women and men in the Bone Microarchitecture International Consortium (BoMIC): a prospective study. Lancet Diabetes Endocrinol.

[14] Johansson L, Sundh D, Zoulakis M, Rudäng R, Darelid A, Brisby H, Nilsson AG, Mellström D, Lorentzon M (2018). The prevalence of vertebral fractures is associated with reduced hip bone density and inferior peripheral appendicular volumetric bone density and structure in older women. J Bone Miner Res.

[15] Boutroy S, Khosla S, Sornay-Rendu E, Zanchetta MB, McMahon DJ, Zhang CA, Chapurlat RD, Zanchetta J, Stein EM, Bogado C (2016). Microarchitecture and peripheral BMD are impaired in postmenopausal white women with fracture independently of total hip T-score: an international multicenter study. J Bone Miner Res.

[16] Zhu TY, Yip BH, Hung VW, Choy CW, Cheng KL, Kwok TC, Cheng JC, Qin L (2018). Normative standards for HRpQCT parameters in Chinese men and women. J Bone Miner Res.

[17] Genant HK, Wu CY, van Kuijk C, Nevitt MC (1993). Vertebral fracture assessment using a semiquantitative technique. J Bone Miner Res.

[18] Hung VW, Zhu TY, Cheung WH, Fong TN, Yu FW, Hung LK, Leung KS, Cheng JC, Lam TP, Qin L (2015). Age-related differences in volumetric bone mineral density, microarchitecture, and bone strength of distal radius and tibia in Chinese women: a high-resolution pQCT reference database study. Osteoporos Int.

[19] Pialat JB, Burghardt AJ, Sode M, Link TM, Majumdar S (2012). Visual grading of motion induced image degradation in high resolution peripheral computed tomography: impact of image quality on measures of bone density and micro-architecture. Bone.

[20] Burghardt AJ, Buie HR, Laib A, Majumdar S, Boyd SK (2010). Reproducibility of direct quantitative measures of cortical bone microarchitecture of the distal radius and tibia by HR-pQCT. Bone.

[21] Liu XS, Zhang XH, Sekhon KK, Adams MF, McMahon DJ, Bilezikian JP, Shane E, Guo XE (2010). High-resolution peripheral quantitative computed tomography can assess microstructural and mechanical properties of human distal tibial bone. J Bone Miner Res.

[22] Odgaard A, Gundersen HJ (1993). Quantification of connectivity in cancellous bone, with special emphasis on 3-D reconstructions. Bone.

[23] van Rietbergen B, Weinans H, Huiskes R, Odgaard A (1995). A new method to determine trabecular bone elastic properties and loading using micromechanical finite-element models. J Biomech.

[24] Solomou G, Damilakis J (2016). Radiation exposure in bone densitometry. Semin Musculoskelet Radiol.

[25] Boutroy S, Bouxsein ML, Munoz F, Delmas PD (2005). In vivo assessment of trabecular bone microarchitecture by high-resolution peripheral quantitative computed tomography. J Clin Endocrinol Metab.

[26] Paggiosi MA, Debono M, Walsh JS, Peel NFA, Eastell R (2020). Quantitative computed tomography discriminates between postmenopausal women with low spine bone mineral density with vertebral fractures and those with low spine bone mineral density only: the SHATTER study. Osteoporos Int.

[27] El Maghraoui A, Rezqi A, Mounach A, Achemlal L, Bezza A, Ghozlani I (2013). Systematic vertebral fracture assessment in asymptomatic postmenopausal women. Bone.

[28] Wang J, Stein EM, Zhou B, Nishiyama KK, Yu YE, Shane E, Guo XE (2016). Deterioration of trabecular plate-rod and cortical microarchitecture and reduced bone stiffness at distal radius and tibia in postmenopausal women with vertebral fractures. Bone.

[29] Sornay-Rendu E, Boutroy S, Duboeuf F, Chapurlat RD (2017). Bone microarchitecture assessed by HR-pQCT as predictor of fracture risk in postmenopausal women: the OFELY study. J Bone Miner Res.

[30] Burt LA, Manske SL, Hanley DA, Boyd SK (2018). Lower bone density, impaired microarchitecture, and strength predict future fragility fracture in postmenopausal women: 5-year follow-up of the Calgary CaMos Cohort. J Bone Miner Res.

[31] Heyer FL, de Jong JJA, Willems PC, Arts JJ, Bours SGP, van Kuijk SMJ, Poeze M, Geusens PP, van Rietbergen B, van den Bergh JP (2019). Long-term functional outcome of distal radius fractures is associated with early post-fracture bone stiffness of the fracture region: an HR-pQCT exploratory study. Bone.

[32] Zhu TY, Hung VW, Cheung WH, Cheng JC, Qin L, Leung KS (2016). Value of measuring bone microarchitecture in fracture discrimination in older women with recent hip fracture: a case-control study with HR-pQCT. Sci Rep.

[33] Vranken L, Wyers CE, van Rietbergen B, Driessen JHM, Geusens P, Janzing HMJ, van der Velde RY, van den Bergh JPW (2019). The association between prevalent vertebral fractures and bone quality of the distal radius and distal tibia as measured with HR-pQCT in postmenopausal women with a recent non-vertebral fracture at the Fracture Liaison Service. Osteoporos Int.

[34] Torres GHF, Guzman LFE, Alvarenga JC, Lopes NHM, Pereira RMR (2019). Association of moderate/severe vertebral fractures with reduced trabecular volumetric bone density in older women and reduced areal femoral neck bone density in older men from the community: a cross-sectional study (SPAH). Maturitas.

[35] Xie F, Zhou B, Wang J, Liu T, Wu X, Fang R, Kang Y, Dai R (2018) Microstructural properties of trabecular bone autografts: comparison of men and women with and without osteoporosis. Arch Osteoporos 13(1):18 10.1007/s11657-018-0422-z 29508160

[36] Seeman E (2008). Structural basis of growth-related gain and age-related loss of bone strength. Rheumatology (Oxford).

[37] Sundh D, Nilsson AG, Nilsson M, Johansson L, Mellström D, Lorentzon M (2017). Increased cortical porosity in women with hip fracture. J Intern Med.

[38] Burghardt AJ, Kazakia GJ, Ramachandran S, Link TM, Majumdar S (2010). Age- and gender-related differences in the geometric properties and biomechanical significance of intracortical porosity in the distal radius and tibia. J Bone Miner Res.

[39] Burghardt AJ, Pialat JB, Kazakia GJ, Boutroy S, Engelke K, Patsch JM, Valentinitsch A, Liu D, Szabo E, Bogado CE (2013). Multicenter precision of cortical and trabecular bone quality measures assessed by high-resolution peripheral quantitative computed tomography. J Bone Miner Res.

[40] Zebaze R, Seeman E (2015). Cortical bone: a challenging geography. J Bone Miner Res.

[41] Bjørnerem Å, Bui M, Wang X, Ghasem-Zadeh A, Hopper JL, Zebaze R, Seeman E (2015). Genetic and environmental variances of bone microarchitecture and bone remodeling markers: a twin study. J Bone Miner Res.

[42] Lee DC, Campbell PP, Gilsanz V, Wren TAL (2009). Contribution of the vertebral posterior elements in anterior-posterior DXA spine scans in young subjects. J Bone Miner Res.

[43] Sanfelix-Genoves J, Arana E, Sanfelix-Gimeno G, Peiro S, Graells-Ferrer M, Vega-Martinez M (2012). Agreement between semi-automatic radiographic morphometry and Genant semi-quantitative method in the assessment of vertebral fractures. Osteoporos Int.

[44] Liu XS, Cohen A, Shane E, Yin PT, Stein EM, Rogers H, Kokolus SL, McMahon DJ, Lappe JM, Recker RR (2010). Bone density, geometry, microstructure, and stiffness: relationships between peripheral and central skeletal sites assessed by DXA, HR-pQCT, and cQCT in premenopausal women. J Bone Miner Res.

